# Chiropractic Management of Lumbar Disc Herniation in a Patient With Co-existing Liver Cancer: A Case Report

**DOI:** 10.7759/cureus.51445

**Published:** 2024-01-01

**Authors:** Eric Chun-Pu Chu, Shun Zhe Piong, Cliff Tao

**Affiliations:** 1 Chiropractic and Physiotherapy Centre, New York Medical Group, Hong Kong, CHN; 2 Chiropractic Department, EC Healthcare, New York Medical Group, Hong Kong, HKG; 3 Radiology, Private Practice of Chiropractic Radiology, Irvine, USA

**Keywords:** chiropractic, chiropractor, lumbar disc herniation, disc herniation, liver cancer

## Abstract

The current case report outlines the chiropractic management of a 30-year-old male construction worker who presented with symptoms of lumbar disc herniation with co-existing stage IV liver cancer. The patient reported experiencing substantial lower back pain and decreased sensation in his right leg following a fall at work, impacting his mobility and quality of life. The complexity of this case is underscored by the challenge of differentiating between pain due to metastatic disease and that related to the fall. The chiropractic treatment plan included gentle joint mobilization, instrument-assisted soft tissue mobilization, and low-impact exercises tailored to the patient’s overall health status. The treatment protocol markedly improved pain levels, range of motion, and overall quality of life. This case highlights the potential role of chiropractic care in managing complex cases of lumbar disc herniation, even in the presence of severe illnesses such as liver cancer. This study provides valuable insights into the importance of personalized and adaptable treatment strategies in managing such cases, contributing a unique perspective to the scientific literature.

## Introduction

Lumbar disc herniation is a musculoskeletal disorder that often results in severe pain and disability [1] and necessitates multifaceted management when complicated by co-existing conditions such as advanced systemic diseases, including cancer. The current case report presented a unique scenario in which a common musculoskeletal disorder intersected with a diagnosis of stage IV liver cancer. The patient was a 30-year-old male construction worker who developed symptoms of lumbar disc herniation following a fall at the workplace.

Patients with cancer have been known to visit chiropractic clinics to address musculoskeletal pain [2-4], and the complexity of managing the current case is heightened by advanced liver cancer, which can substantially influence both the therapeutic approach and the patient's response to treatment. Careful differentiation is required to determine whether the reported leg pain originates from metastatic disease or fall-related musculoskeletal disorder pain [2-4]. Moreover, advanced cancer can potentially limit the application of certain chiropractic procedures owing to systemic frailty, risk of fractures from potential metastasis, and poor overall health status. The herniation-induced pain can be further compounded by the discomfort associated with advanced liver cancer, adding another layer of complexity to symptom management.

To the best of our knowledge, this is the first study to explore the chiropractic management of lumbar disc herniation in a patient with co-existing liver cancer. Our findings could offer novel insights and guidance for clinicians encountering similar cases and underscore the potential for chiropractic care to improve quality of life, even in patients with advanced systemic diseases.

## Case presentation

**Figure 1:**
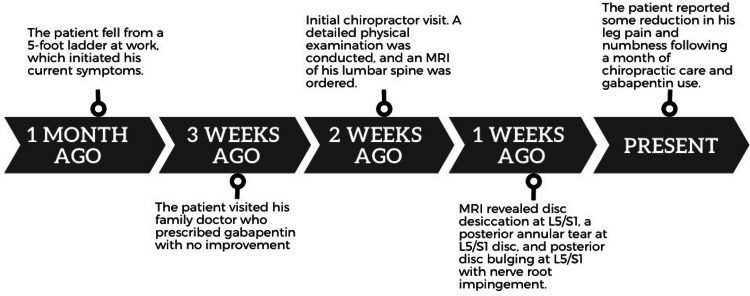
Timeline of care

A 30-year-old male construction worker with a known history of stage IV liver cancer presented to a chiropractor with a one-month history of constant right leg pain and numbness (Figure 1). The pain, described as a shooting sensation starting from his lower back to his left leg, was exacerbated by walking and standing for prolonged periods. Based on the Numeric Pain Rating Scale, the pain was graded as 7/10. The symptoms commenced after a fall from a 5-foot ladder at work, which substantially interfered with his daily activities. He was unable to perform work-related activities, had difficulty sleeping, and experienced a reduction in his overall physical activity. The patient was examined by a family physician immediately after the fall. The patient was prescribed gabapentin at a dose of 300 mg three times a day for neuropathic pain control. However, this medication provided only minimal improvement during the course of three months. His quality of life was severely impacted (48%), as assessed using the World Health Organization Quality of Life (WHOQOL) scale. Subsequently, he sought chiropractic consultation for low back pain.

**Figure 2:**
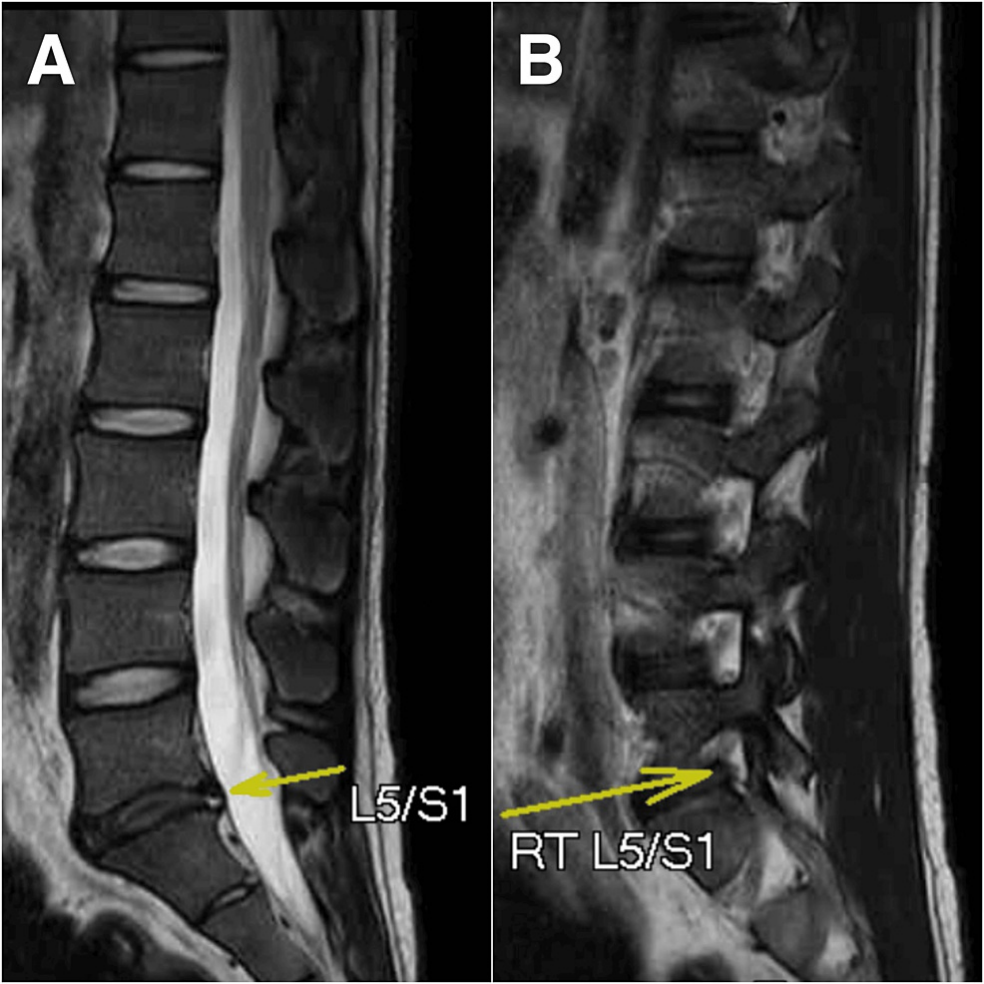
Sagittal view of magnetic resonance image (MRI) of lumbar spine A) MRI showing disc desiccation with decreased disc height noted at L5/S1 level (yellow arrow). A posterior annular tear can be observed at the L5/S1 disc. Disc desiccation with decreased disc height at L5/S1 level. A posterior annular tear is observed at the L5/S1 disc. B) Posterior disc bulging at L5/S1 level with impingement onto right S1 descending nerve root (yellow arrow).

**Figure 3:**
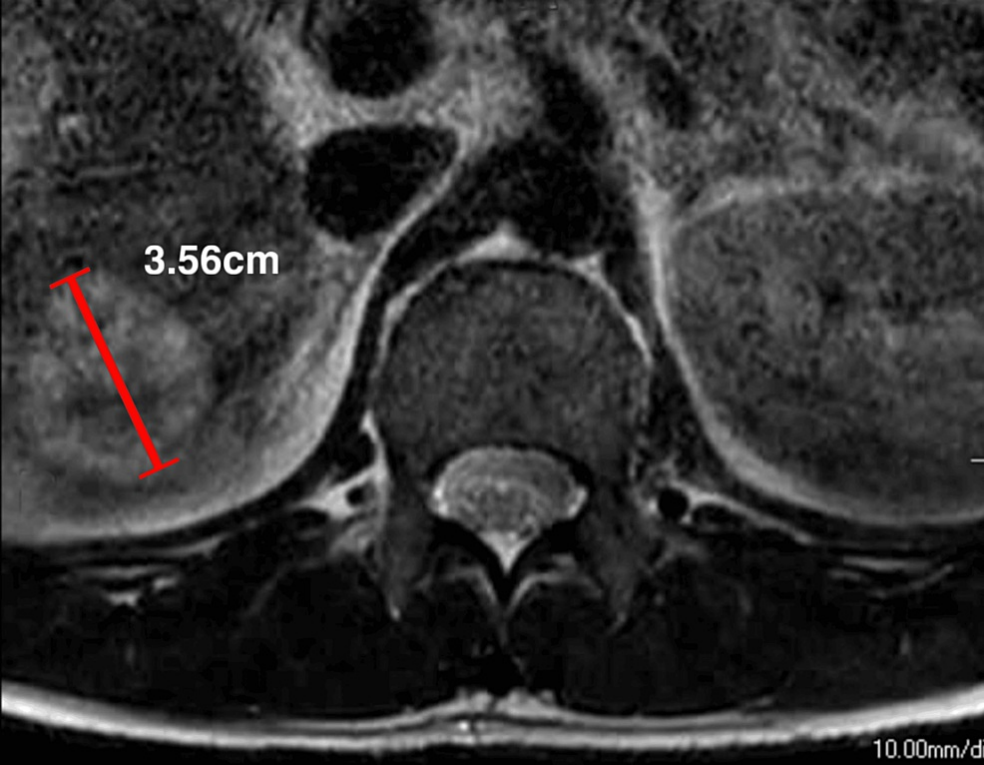
Axial view of magnetic resonance image (MRI) of lumbar spine Multiple liver lesions measuring up to 3.56 cm are seen, indicative of liver metastases.

Upon examination, he presented with a pronounced limp and decreased sensation to light touch and pinpricks in his left lower extremities. The straight leg-raise test result was positive at 45° on the left side. The range of motion in the lumbar spine was recorded using a goniometer, which was reduced by approximately 50% in all directions owing to pain and stiffness. Given the patient's history of liver cancer, differentiating whether the leg pain was due to metastatic disease or related to a fall was challenging. Magnetic resonance imaging was performed to confirm the diagnosis by the chiropractor. The findings revealed disc desiccation with decreased disc height at the L5/S1 level, posterior disc bulging at the L5/S1 level (Figure 2A) with impingement of the left S1 (Figure 2B) descending nerve root, and posterior annular tear at the L5/S1 disc. As expected, multiple liver lesions measuring up to 3.56 cm are seen, indicative of liver metastases (Figure 3).

**Figure 4:**
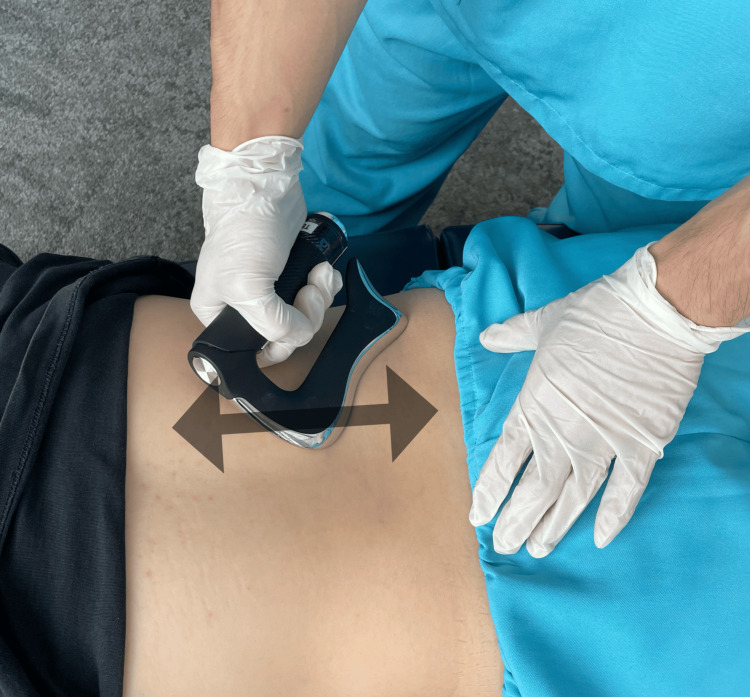
Instrument-Assisted Soft Tissue Mobilization (IASTM) technique IASTM involves specialized tools to detect and treat fascial restrictions, reducing muscle tightness and inflammation around the lumbar area.

A chiropractic treatment plan was carefully designed to manage the lumbar disc herniation while considering the co-existing stage IV liver cancer. The chiropractor subjected the lumbar spine to gentle, low-force joint mobilization to increase flexibility, reduce pain, and improve range of motion. In addition, instrument-assisted soft tissue mobilization (IASTM) was performed, which involves specialized tools to detect and treat fascial restrictions, thereby reducing muscle tightness and inflammation around the lumbar area (Figure 4). The third component of the treatment plan involved a series of low-impact exercises aimed at strengthening the muscles and improving coordination of the left lower limb. Combining in-clinic treatments with at-home exercise and Tai Chi, the treatment plan aimed to optimize the patient's mobility and reduce pain levels while bolstering his overall physical function. The patient complied with the recommended treatment and tolerated the interventions well, with no reported adverse effects.

After one month of chiropractic care and gabapentin use, the patient reported a reduced pain level of 1/10. The range of motion of his lumbar spine showed considerable improvement, and his walking distance increased from 100 to 800 m without pain. According to the WHOQOL scale, the patient’s quality of life improved to 90% (Table 1). However, the patient still experienced poor sleep quality owing to persistent back pain.

**Table 1:** Comparison of pre- and post-treatment results

	Pain intensity	Quality of life	Walking distance
Pre-treatment	7/10	48%	100 meter
Post-treatment	1/10	90%	800 meter

No adverse events occurred during chiropractic care. The patient will continue his care with the chiropractor and oncologist, with close monitoring of his liver function and the progression of liver cancer.

## Discussion

In the current case report, the treatment protocol underscores the potential role of chiropractic care in managing conditions such as lumbar disc herniation, even in patients with advanced systemic diseases such as liver cancer. The comprehensive treatment plan, which incorporated gentle, low-force joint mobilization, IASTM, and a series of low-impact exercises, markedly improved the patient's pain level, healing, range of motion, and overall quality of life [5,6]. Despite the complexity of the patient’s health status, these positive outcomes highlight the effectiveness of chiropractic intervention.

Differentiating between pain due to metastatic disease and pain related to lumbar disc herniation presents a unique challenge [7]. To address this issue, we adopted a multidimensional approach. First, we closely monitored the patient’s pain patterns, noting any changes in intensity, frequency, and location. We also maintained open communication with the patient’s oncologists to understand cancer progression and its potential impact on pain levels. Diagnostic imaging is crucial in identifying new or progressive metastatic lesions that could contribute to pain. By integrating these strategies, we could substantially differentiate between herniation-induced pain and pain attributed to metastatic disease.

Given the patient's advanced liver cancer, chiropractic procedures were cautiously performed. High-velocity, low-amplitude techniques were avoided owing to the potential risk of bone metastasis-induced pathologic fractures [8]. Likewise, we refrained from applying deep pressure during manual therapy to avoid aggravating existing liver pain or discomfort.

Importantly, the current case has broader implications for chiropractic management of similar cases in the future, underlining the necessity for a careful, individualized approach that considers the patient's overall health status and co-existing conditions. Moreover, our findings emphasize the importance of closely monitoring the patient's response to treatment and adjusting the treatment plan as required. Chiropractic care can complement traditional medical treatments in managing pain and improving the quality of life of patients with advanced systemic diseases [9,10].

The patient-centered and adaptable approach was valuable in the current case report. The treatment plan was designed considering the patient's specific needs and limitations and was adjusted based on his response to treatment. The use of various chiropractic techniques provided a comprehensive approach to symptom management, while the incorporation of at-home exercises empowered the patient to actively participate in his own care. However, managing a patient's poor sleep quality due to persistent back pain remains a challenge that warrants additional attention. Future studies should consider incorporating strategies to improve sleep quality, such as sleep hygiene education or referral to sleep specialists.

## Conclusions

This unique case of a 30-year-old male with co-existing stage IV liver cancer and lumbar disc herniation provides invaluable insights into the potential role of chiropractic care in managing complex musculoskeletal issues alongside severe systemic diseases. Our results indicate the potential benefits of an adaptable, patient-centered chiropractic approach, even in challenging clinical contexts. Furthermore, the observed results highlight the need for further research to validate these findings and explore the integration of chiropractic care into comprehensive treatment plans for patients with similar conditions. Notably, the current case report underscores the importance of inter-professional collaboration and communication in managing similar complex cases. Moreover, differentiating between pain due to metastatic disease and musculoskeletal issues remains a considerable challenge, warranting the need for future studies to develop more effective strategies. Poor sleep quality remains a persistent limitation that should be addressed to enhance overall patient care. The findings of the current case can serve as a springboard for the chiropractic community to manage similar scenarios, emphasizing the importance of caution, adaptability, and a comprehensive patient-centered approach.
